# Graphical analysis of NMR structural quality and interactive contact map of NOE assignments in ARIA

**DOI:** 10.1186/1472-6807-8-30

**Published:** 2008-06-05

**Authors:** Benjamin Bardiaux, Aymeric Bernard, Wolfgang Rieping, Michael Habeck, Thérèse E Malliavin, Michael Nilges

**Affiliations:** 1 Unité de Bioinformatique Structurale, CNRS URA 2185, Institut Pasteur, 28, rue du Dr. Roux, 75015 Paris, France; 2 Dept of Biochemistry, University of Cambridge, 80 Tennis Court Road, Cambridge CB2 1GA, UK; 3 Max Planck Institute for Developmental Biology, Spemannstrasse 35 and Max Planck Institute for Biological Cybernetics, Spemannstrasse 38, 72076 Tübingen, Germany

## Abstract

**Background:**

The Ambiguous Restraints for Iterative Assignment (ARIA) approach is widely used for NMR structure determination. It is based on simultaneously calculating structures and assigning NOE through an iterative protocol. The final solution consists of a set of conformers and a list of most probable assignments for the input NOE peak list.

**Results:**

ARIA was extended with a series of graphical tools to facilitate a detailed analysis of the intermediate and final results of the ARIA protocol. These additional features provide (i) an interactive contact map, serving as a tool for the analysis of assignments, and (ii) graphical representations of structure quality scores and restraint statistics. The interactive contact map between residues can be clicked to obtain information about the restraints and their contributions. Profiles of quality scores are plotted along the protein sequence, and contact maps provide information of the agreement with the data on a residue pair level.

**Conclusion:**

The graphical tools and outputs described here significantly extend the validation and analysis possibilities of NOE assignments given by ARIA as well as the analysis of the quality of the final structure ensemble. These tools are included in the latest version of ARIA, which is available at . The Web site also contains an installation guide, a user manual and example calculations.

## Background

The calculation of an NMR (Nuclear Magnetic Resonance) structure is most often realised in parallel with the assignment of NOEs (Nuclear Overhauser Effect). This task can be automatically performed in the software ARIA (Ambiguous Restraints for Iterative Assignment) [1,2]. The ARIA program uses the concept of Ambiguous Distance Restraints [3] to convert multiple assignment possibilities for an NOE into a single restraint. An iterative protocol allows to identify unlikely assignments and noise peaks to progressively reduce the ambiguity and clean the dataset.

In the first iteration, all assignments that are consistent with the chemical shift assignment are applied to the structure. In each iteration, the current set of restraints is used to generate a structure ensemble. Statistics are performed after each iteration on each possible assignment and on how often each restraint is violated as a whole. The least likely assignment possibilities, and systematically violated restraints are removed. This results in a restraint list with fewer possibilities per restraint, and where the restraints that most likely correspond to noise peaks are removed. After the last iteration, the best energy structures are refined using a short molecular dynamics run in water [4].

The current state of the protocol including ambiguous assignments and distance violations is summarised in several report files located in each iteration directory. Analysing such text files is difficult since they contain a large number of data. ARIA was thus extended to allow the generation of an interactive contact map, which provides a detailed analysis of the restraints and restraint contributions.

Analysing the quality of NMR structure is a key step into the validation of an ARIA calculation. In that respect, it was recently shown [5] that profiles of quality scores calculated on individual residues along the biomolecular structure can be essential to detect possible sources of error in the spectral assignment. Several extensions of ARIA were therefore implemented in order to generate postscript files describing the structural quality and the restraint violations at the residue level.

## Implementation

ARIA is written in the programming language Python. The version 2.2 of ARIA now also supports the Python extensions package Numpy [6] for computationally intensive matrix operations. Numpy is meant to replace the older Numeric package. Both packages employ optimised C and Fortran libraries such as BLAS. Additionally, ARIA 2.2 requires the Matplotlib [7] module to plot graphics during the analysis. For setting-up a project, ARIA offers a graphical user interface (GUI) written in Python and based on the Tcl/Tk and Tix graphics libraries. The modular and highly object-oriented design of the program facilitates the addition of new features, such as the ones presented here.

### Interactive peak maps

In each iteration, the current assignments are stored in the form of a binary file that can be analysed afterwards. An additional section in the GUI provides a way to read back the assignments and display them as a clickable contact map. This map is defined as a Tk canvas widget and each pixel is clickable to present additional information about this particular contact. A pop-up window displays the corresponding assignments in tables that can be exported as text files. The peak map can be saved in Postscript format.

### Quality profiles

Postscript files describing RMS (Root Mean Square) differences from distance bounds and individual WHATIF scores along the sequence are automatically created at the end of each iteration or after the final structure analysis. The graphics are plotted with the matplotlib plotting library interfaced with Python. Quality and RMS profiles data are also stored in formatted text files for further use.

## Results and Discussion

### Interactive analysis of peaks assignments

For each ARIA iteration, the interactive peak map displays the pairs of residues involved in one or more assignment possibilities. Such maps can be generated from the current state of the assignment with three classes of restraints: (i) all restraints, (ii) ambiguous restraints and (iii) unambiguous restraints.

**Figure 1:**
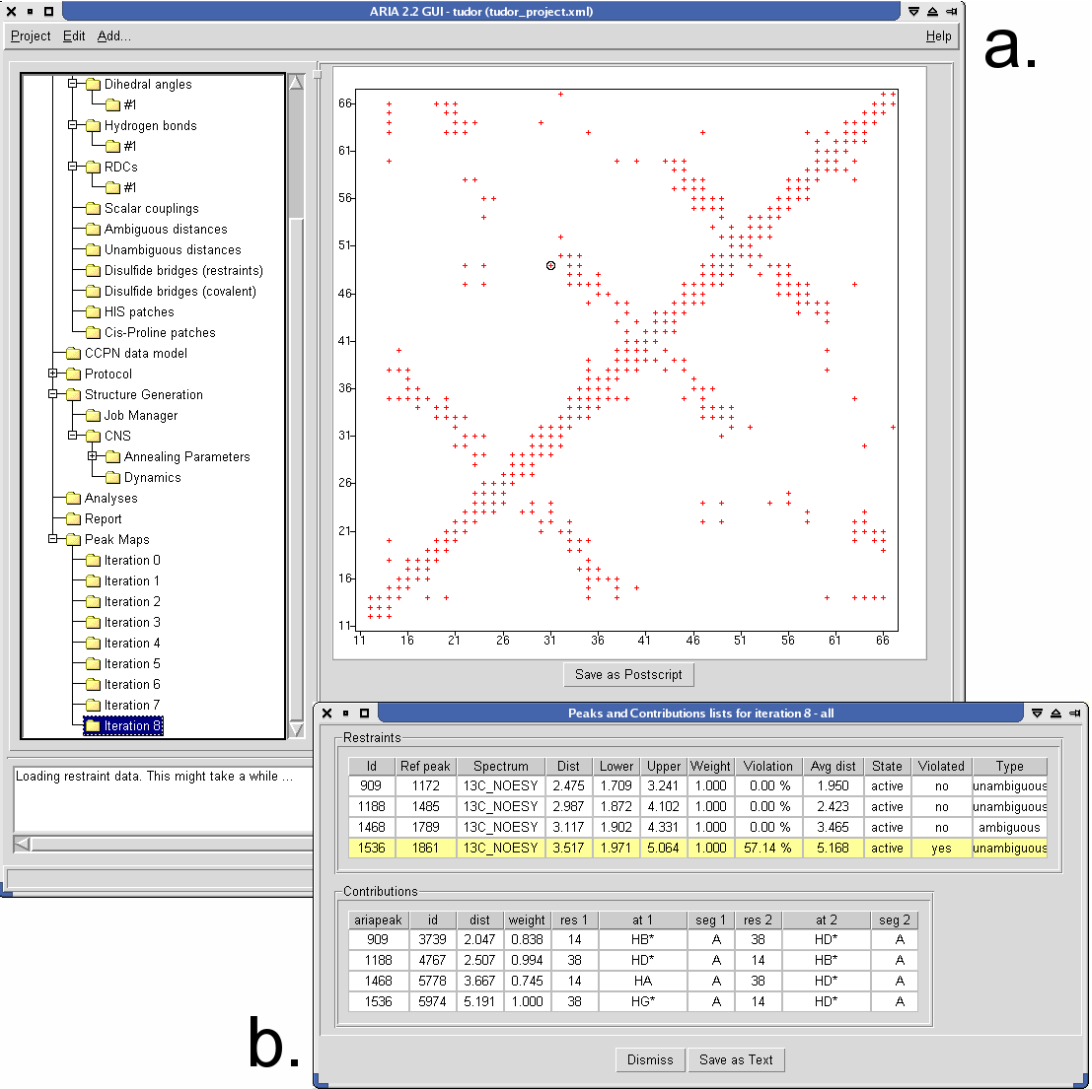
**Interactive peak map**. (a) Right panel of the ARIA 2.2 GUI showing the interactive peak map at the iteration 8 of an ARIA run. Each pixel of the map located between residues *i *and *j *is clickable and an assignment report (b) can be opened, containing the list of ARIA peaks existing between the residues *i *and *j*, along with their contributions.

Clicking on a pixel located at the position (*i*, *j*) on the map (Figure 1a) opens a pop-up window (Figure 1b) that shows a list of ARIA restraints involving atoms from residues *i *and *j*. It also gives information about each assignment possibility ("contribution") of these restraints. Multiple pixel selection is possible.

The restraints lists are displayed in tables, indicating different parameters such as the target distance, the percentage of structures in which a restraint was violated or the average distance found in the structure ensemble. A colour code indicates whether a restraint is globally violated. For each assignment possibility, the table indicates the relative weight, the effective distance as well as the description of the pairs of atoms involved.

This interactive tool allows the user to get a detailed analysis of the peak assignment procedure at each step of the ARIA protocol. Since the results are presented as a two dimensional map, this tool significantly extends the information content with respect to the standard ARIA reports. Moreover, the dynamic and graphical nature of the map may allow a rapid detection of the possible errors in the assignment process, or of the potential inconsistencies in the data.

### Per-residue structural quality

Postscript files describing (i) the restraints, through the RMS of deviations from the distance bounds, and (ii) the structure quality, through the WHATIF [8] scores, are generated automatically during a run. These parameters are displayed at the residue level, in the form of a profile along the protein sequence, or as a contact map for the RMS deviations per residue pair.

**Figure 2:**
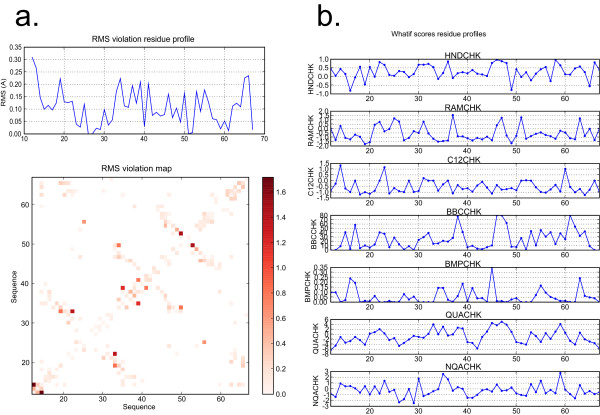
**Per-residue quality plots**. (a) Contact map displaying the sums of RMS deviations and profile of the RMS deviations (b) WHATIF score profiles along the sequence. The RMS deviations are plotted with a colour scale.

The contact map displays the sum of the RMS deviations (Figure 2a) per residue pair. In the profiles, the sum of the RMS of violations per residues and the mean values over the conformers of the WHATIF scores are plotted along the protein sequence (Figure 2b). The most informative WHATIF scores are plotted, such as the packing quality Z-score (1st and 2nd generation), inter-atomic bumps as well as the backbone conformation Z-score.

An essential part in the validation of an ARIA calculation is the analysis of the quality of the NMR structures. Classically, the overall number of violations or the RMS deviations in addition to global WHATIF scores of the whole molecule are used to assess the quality of a structure. In the light of recent investigations [5], it is clear that these global parameters may not suffice to readily detect errors in the local or global fold of a protein. The analysis of quality scores of each residue along the molecular sequence is essential to precisely detect possible sources of error in the spectral assignment. The automated generation of per-residue profiles for RMS deviations and for WHATIF scores provides a highly integrated tool to rapidly identify regions of the structure that exhibit abnormal quality factors, and where restraints and assignments should be more thoroughly investigated.

## Conclusion

The graphical tools described here represent a significative extension of the possibilities to analyse NOE assignments and the quality of the solution given by ARIA. The tools were developed to allow an analysis at the residue level in an interactive way, which is critical for the assessment of the solution and the detection of errors.

## Availability and requirements

Project name: ARIA 2.2

Project home page: 

Operating system(s): Linux, Mac OS X, SGI

Programming language: Python

Other requirements: Numpy, Tcl/Tk, ScientificPython

License: no license required.

## Authors' contributions

BB implemented the graphical tools of ARIA 2 and helped to draft the manuscript. AB participated in programming the tools. MN conceived ARIA, WR and MH implemented the version 2 of the program. TEM drafted the manuscript. All authors read and approved the final manuscript.
